# Landscape reveals critical network structures for sharpening gene expression boundaries

**DOI:** 10.1186/s12918-018-0595-5

**Published:** 2018-06-13

**Authors:** Chunhe Li, Lei Zhang, Qing Nie

**Affiliations:** 1 Shanghai Center for Mathematical Sciences, Fudan University, Shanghai, 200433 China; 2 Institute of Science and Technology for Brain-Inspired Intelligence, Fudan University, Shanghai, 200433 China; 3 Beijing International Center for Mathematical Research, Peking University, Beijing, 100871 China; 4 Center for Quantitative Biology, Peking University, Beijing, 100871 China; 5 Department of Mathematics, University of California, Irvine, 92697 USA; 6 Center for Complex Biological Systems, University of California, Irvine, 92697 USA

**Keywords:** Gene expression boundaries, Morphogen, Landscape, Switching time

## Abstract

**Background:**

Spatial pattern formation is a critical issue in developmental biology. Gene expression boundary sharpening has been observed from both experiments and modeling simulations. However, the mechanism to determine the sharpness of the boundary is not fully elucidated.

**Results:**

We investigated the boundary sharpening resulted by three biological motifs, interacting with morphogens, and uncovered their probabilistic landscapes. The landscape view, along with calculated average switching time between attractors, provides a natural explanation for the boundary sharpening behavior relying on the noise induced gene state switchings. To possess boundary sharpening potential, a gene network needs to generate an asymmetric bistable state, i.e. one of the two stable states is less stable than the other. We found that the mutual repressed self-activation model displays more robust boundary sharpening ability against parameter perturbation, compared to the mutual repression or the self-activation model. This is supported by the results of switching time calculated from the landscape, which indicate that the mutual repressed self-activation model has shortest switching time, among three models. Additionally, introducing cross gradients of morphogens provides a more stable mechanism for the boundary sharpening of gene expression, due to a two-way switching mechanism.

**Conclusions:**

Our results reveal the underlying principle for the gene expression boundary sharpening, and pave the way for the mechanistic understanding of cell fate decisions in the pattern formation processes of development.

**Electronic supplementary material:**

The online version of this article (10.1186/s12918-018-0595-5) contains supplementary material, which is available to authorized users.

## Background

A persisting focal issue in developmental biology is how the spatial pattern of gene expression is formed. During embryonic development, cells with different expression patterns occupy different domains separated by sharp borders. It is suggested that cell exploits the diffusive molecules and morphogens to receive positional information [1–3]. Morphogens, by forming concentration gradients, specify distinct cell fates and regulate patterns. So, cell fate decisions are vital for the pattern formation in the developmental process [4–6]. The challenge is to understand how cells form sharp gene expression boundaries from light morphogen gradients. Some mechanisms involve cell sorting resulting from cell movements and differential adhesion [7, 8]. Also, some cell-intrinsic boundary-forming mechanisms, which transfer the differences in morphogen gradients into sharp change in downstream gene expression, have been proposed [9]. However, the mechanisms for boundary formation and sharpening remain not fully described.

In cells, there are intrinsic fluctuations from limited number of molecules and external fluctuations from inhomogeneous environments [10–14]. Therefore, the gene expression fluctuations need to be considered to simulate the real cellular environments. Some noise attenuation mechanisms have been suggested in cell fate decision processes during developmental patterning [15, 16]. Recently, Zhang et al. proposed a noise induced cell fate switching mechanism to explain gene expression boundary sharpening in the zebrafish hindbrain [15]. In their work, noise has been shown to play some critical roles in developmental pattern formation processes by facilitating gene expression state switchings. However, the underlying mechanism for how the noise promotes the sharpening of gene expression borders remains elusive.

In this work, we constructed a mutual repressed self-activation (MRSA) model, which interacts with morphogens. The involved two genes (X and Y) are both activated by a morphogen M. In particular, we introduce the fluctuations to both morphogen gradients and gene expression levels. To explore the boundary sharpening mechanisms related to gene state transitions, we resort to a probabilistic landscape approach [17–19], which has been employed to study the stability of attractors for the gene networks and the switchings between the attractors. The landscape was proposed by Waddington to characterize development and differentiation of cells, as a metaphor [20]. From the landscape theory, different phenotypes can be depicted as the basins of attraction on a potential landscape surface, and the cell fate decision process can be viewed as a ball rolling down from one basin to another on the landscape surface.

We mapped out the landscape for the mutual repressed self-activation model at different spatial locations (corresponding to different Morphogen levels). At the position close to the gene expression boundary, the landscape displays an unbalanced bistable state, where one attractor is much less stable than the other. The landscape view for the system transforms the boundary sharpening problem to the understanding of cell fate transitions between basins, and thus provides a natural explanation for the noise induced boundary sharpening behavior. We compared three common motifs - mutual repressed self-activation, mutual repression, and self-activation - to see the effects of network topology on the boundary sharpening ability. We found that mutual repressed self-activation model has the better boundary sharpening performance against fluctuations, which is consistent with the results that mutual repressed self-activation model possesses the shorter switching time calculated from the landscape among three models. Adding a second morphogen to the models provides more stable boundary sharpening ability due to a two-way switching mechanism. Our results reveal the underlying mechanisms for noise induced boundary sharpening of gene expression, and shed light on the deeper understanding of spatial pattern formation in embryonic development.

## Results

### Landscape explains the gene expression boundary sharpening from a mutual repressed self-activation model

**Fig. 1:**
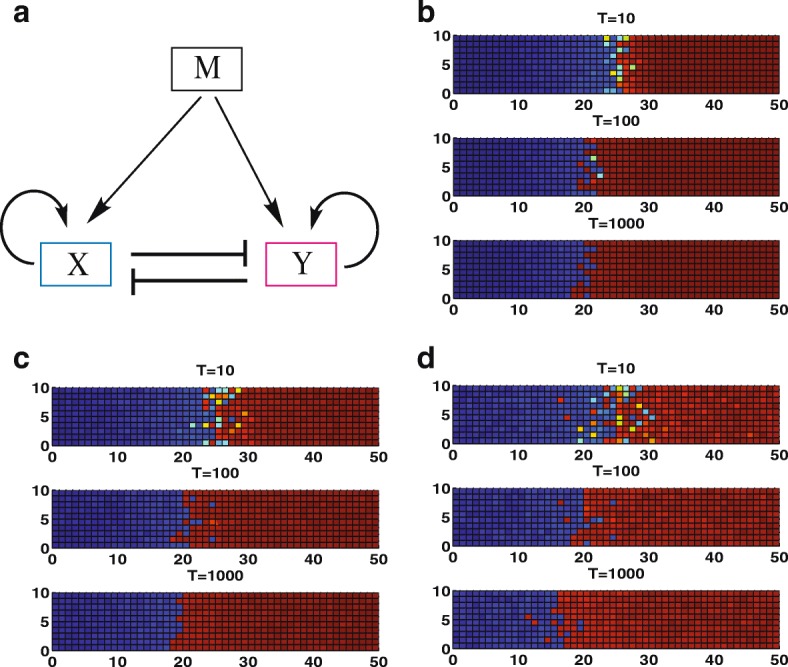
Diagram and two dimensional simulations for boundary sharpening. **a** Diagram for the mutual repression with self-activation model. **b-d** Two dimensional simulations show the boundary sharpening effects over time at different gene expression noise level *d* (coefficient of variance) (**b** for *d* = 0, **c** for *d* = 0.01, **d** for *d* = 0.02). Blue: X is expressed, red: Y is expressed

We first investigate a mutual repressed self-activation model (MRSA), interacting with a morphogen M (Fig. 1a). In this circuit, gene X and gene Y mutually repress each other and self-activate themselves. The morphogen M activates the expression of both gene X and gene Y. Figure 1b-d show the boundary sharpening effects over time at different gene expression noise level *d* (coefficient of variance) from two-dimensional simulations (A for *d* = 0, B for *d* = 0.01, C for *d* = 0.02). Here each cell is represented by a grid square. The blue grids represent the cells with gene X expressed, and the red grids represent cells with gene Y expressed. For the system without gene expression noise (Fig. 1b), the expressions for gene X and Y generate the rough boundary, due to the fluctuations in the morphogen M. If the noise level is too high (Fig. 1d), the gene expression boundary is also rough because the large fluctuations in the gene expression level dominate the expression states and blur the boundary. Surprisingly, when the noise level is in a certain medium range, the boundary becomes sharpening over time (Fig. 1c). The gene expression noise induced the state transition from X expressed state (blue) to Y expressed state (red). Therefore, around the boundary area most of cells are switched to Y state, which makes the boundary sharpened.

**Fig. 2:**
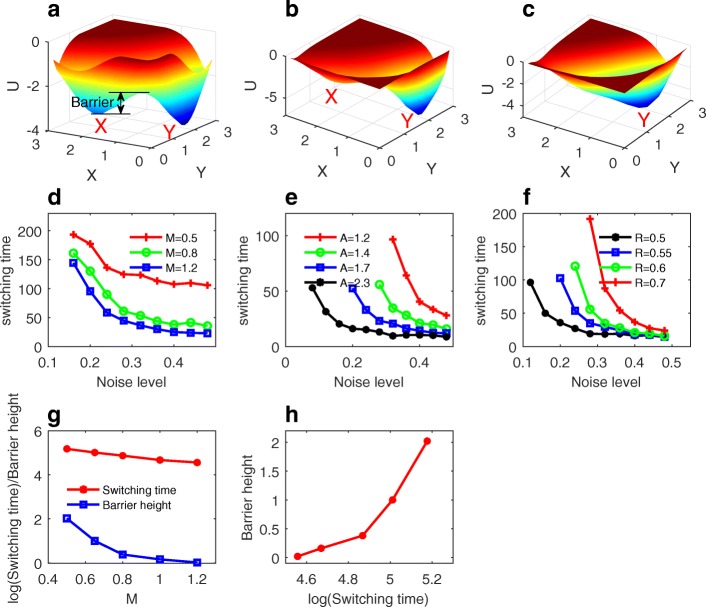
Landscape, barrier heights and switching time. **a-c** Landscapes at different morphogen level M (M = 0.5, 0.8 and 1.2 for **a**, **b** and **c**) for the mutual repression with self-activation (MRSA) model. With M increased, left basin becomes more and more shallow, and therefore the switching from left basin to right basin become more and more easy. **d-f** Average switching time versus noise level separately for different M level (**d**), different A (**e**), and different R (**f**). Every curve (color) is for one parameter set. In (**e**), the parameter A represents the ratio of the synthesis rate for the two genes, which measures the degree of the asymmetry of the system. Other parameters are set as: k = 0.7, b = 0.7, *R* = 0.6. In (**f**), the parameter R represents the strength of the mutual inhibition. Other parameters are set as: k = 0.7, b = 0.7, A = 1.4. **g** Switching time and barrier heights change with morphogen level M. **h** Barrier heights versus the switching time.

To elucidate the mechanism of how noise induces gene state switchings, we acquired the probabilistic landscape at different spatial positions (corresponding to different M level). Landscape is defined as *U* =  −  *log* (*P*_*ss*_) [17, 18, 21–23], and *P*_*ss*_ is the steady state probability distribution (see methods for how to obtain landscape). Figure 2a-c show the probabilistic landscape for the expression level of gene X and Y for the MRSA model at different M levels. Here, the blue region represents the higher probability or lower potential state (attractors), and the red region represents the lower probability or higher potential state. Two stable states (basins) appear on the landscape (bistability). One of them is X expressed state (labeled by X) and the other one is Y expressed state (labelled by Y). The shape of landscape determines the transition difficulty between attractors (X and Y). To quantify the transition difficulty, we define the barrier height (Fig. 2a) as the potential difference from the local minimum (say X attractor) to the saddle point between X attractor and Y attractor. The larger the barrier height, the more difficult the system switches from X state to Y state. Figure 2a-c show that the landscape shape changes as the morphogen M level increases. When M level is 0.5 (Fig. 2a), the landscape exhibits a bistable shape (two basins). As the M increases, the left basin (X attractor) becomes shallower gradually relative to the right basin (Y attractor), and the barrier for X attractor becomes lower, as shown in Fig. 2b. When M increases further (Fig. 2c), the landscape eventually becomes a monostable state (only the Y attractor exists).

The landscape change provides an explanation for the boundary formation and the boundary sharpening effects of MRSA circuit (Fig. 1). When M level is high (Fig. 2c), only Y attractor is stable as indicated on the landscape, so cells will end in the Y expression state (red grids in Fig. 1c). When M level is low (Fig. 2a), gene expression of X and Y form a bistable state, and cells will end in X expression state when assuming gene X is initially expressed [15]. For the medium M level (the central position), landscape also exhibits a bistable state, but X attractor is much less stable than the Y attractor. Induced by certain level of fluctuations, cells will switch from X attractor to Y attractor, which explains why the gene expression boundary will be sharpened. As shown in Fig. 1c, around the boundary position the blue grids change to red grids as T increases from 10 to 1000, which sharpens the boundary. The landscapes obtained here also explain why the noise level has to be in certain range for inducing the boundary sharpening. That is because if the noise level is low it is hard to trigger the switchings from X attractor to Y attractor (cell can not escape from X basin). But if the noise level is too high, the switchings can happen in either direction, meaning that cells can change state from X to Y (jump from X basin to Y basin) but can also change from Y to X (jump from Y basin to X basin). In that case, the boundary sharpening will not take place.

During the development of rhombomeres in the zebrafish hindbrain, the morphogen retinoic acid (RA) induces expression of gene hoxb1a in rhombomere 4 (r4) and gene krox20 in r3 and r5. Fluorescent in situ hybridization reveals rough edges around these gene expression domains, in which cells co-express hoxb1a and krox20 on either side of the boundary, and these sharpen within a few hours [15]. It is found that the boundary sharpening effects are not clear in the r3/r4 boundary from a similar mathematical model [15]. The landscape picture we acquired here provides an explanation for this phenomenon. The r3/r4 boundary is corresponding to the low level M position in our model, which is corresponding to a bistable landscape (Fig. 2a). Here, both two basins are stable, not like the case in the Fig. 2b in which one basin is much less stable than the other. The noise induced transition will not help the sharpening of the boundary because the switching is bidirectional. Cell can switch from X basin to Y basin or from Y basin to X basin. But from population level, the cell type switchings are averaged out, and will not lead to sharpened boundaries.

To further explore the gene state switching mechanisms, we calculated the mean first passage time (MFPT) from state X to state Y, characterizing the average switching time. Now that the boundary sharpening problem has been transformed to a cell fate transition problem, we suppose that the boundary sharpening time should be correlated to the gene state switching time. We calculated the MFPT from the trajectories based on the solution of stochastic ordinary differential equations (see methods). The parameter A represents the ratio of the synthesis rate for the two genes, which measures the asymmetry level of the system. The parameter R represents the strength of the mutual inhibition between gene X and Y. Figure 2d-f show the average switching time from X state to Y state at different noise level, separately for different M, different A, and different R. Here, every curve (color) represents one set of parameter value. Each curve shows that given a noise level what is the average switching time or given a time interval how large noise is needed to trigger the switchings. As the noise level increases, the average switching time declines (Fig. 2d-f), because larger noise promotes the transition between attractors. Also, with M level increased the average switching time decreases (Fig. 2d), i.e., the switching from X state to Y state becomes faster. This is consistent with the landscape analysis because, as M increases, X (Y) attractor becomes less and less (more and more) stable (Fig. 2c), making the transition from X to Y easier. Figure 2e and f show that as the asymmetry level A increases or as the mutual repression strength R decreases, the switching from X state to Y state becomes faster. This indicates that a stable gene state switching for the MRSA model requires larger asymmetry between gene X and Y, and smaller mutual repression strength between gene X and Y. Therefore a natural prediction from these results is that the boundary sharpening will be faster for a circuit with more asymmetry or with weaker mutual repression.

In Fig. 2g and h we compared the MFPT and the barrier heights at different M level. The barrier height quantifies the change of the landscape topography (Fig. 2a-c). The barrier height increases as the switching time increases (Fig. 2h), because a larger barrier makes the switching from X attractor to Y attractor more difficult and thus a longer switching time.

**Fig. 3:**
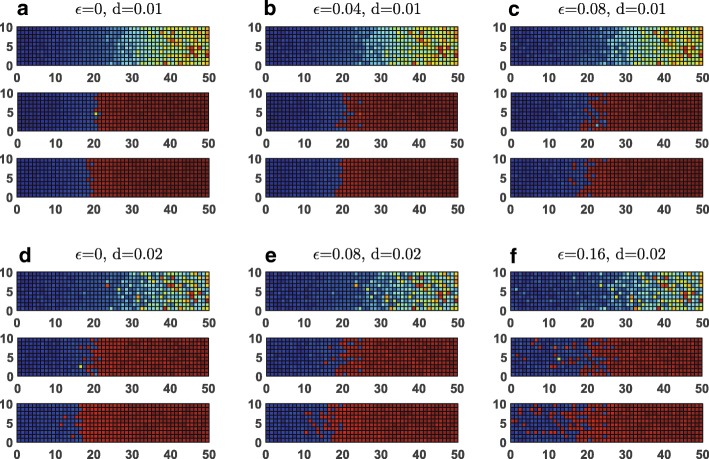
Two dimensional simulations for the boundary sharpening effects over time at different gene expression noise level *d* and different morphogen noise level ϵ for the MRSA model. We define *L* = *ϵ*/*d* as the amplitude ratio between morphogen noise level and gene expression noise level. **a-c** show the simulation results for fixed *d* = 0.01 but *L* increased (**a** for *L* = 0, **b** for *L* = 4 and **c** for *L* = 8). **d-f** show the simulation results for fixed d = 0.02 but *L* increased (**a** for *L* = 0, **b** for *L* = 4 and **c** for *L* = 8). Blue: X is expressed, red: Y is expressed

We also calculated the results for different amplitude ratio (*L* = *ϵ*/*d*) between morphogen noise level *ϵ* and gene expression noise level *d* for MRSA model. As we can see from Figs. 1 and 3, the gene expression noise level *d* has to be in an appropriate range to generate boundary sharpening effects. If the gene expression noise level *d* is too big, the boundary sharpening effects will not occur no matter how we choose the value of morphogen noise level or the amplitude ratio *L* (Fig. 3d-f). Given an appropriate value of *d* (Fig. 3a-c, *d* = 0.01), the morphogen noise level or the amplitude ratio *L* also needs to be tuned. If the morphogen noise level or the amplitude ratio *L* is too big, boundary is not sharpened (Fig. 3c). If the morphogen noise level or the amplitude ratio *L* is too small, the boundary keeps sharpened most of the time except for a quick transition period from initial uneven distributions (Fig. 3a). We also provide the quantitative comparisons of boundary sharpening effects over time from sharpening index (SI), for MRSA models at different morphogen noise level ϵ and gene expression noise level d value (Additional file 1: Figure S1), which provide quantitative support to our above conclusions.

To see how the vertical resolution influence the effects of the boundary sharpening, we also double the number of the vertical grids and made the simulations for the MRSA model (Additional file 1: Figure S2). Our results show that increasing the grid number in the vertical direction does not influence the major conclusions (Additional file 1: Figure S2).

### Mutual repressed self-activation (MRSA) model is more robust for generating gene expression boundary sharpening than self-activation (SA) model or mutual-repression (MR) model

**Fig. 4:**
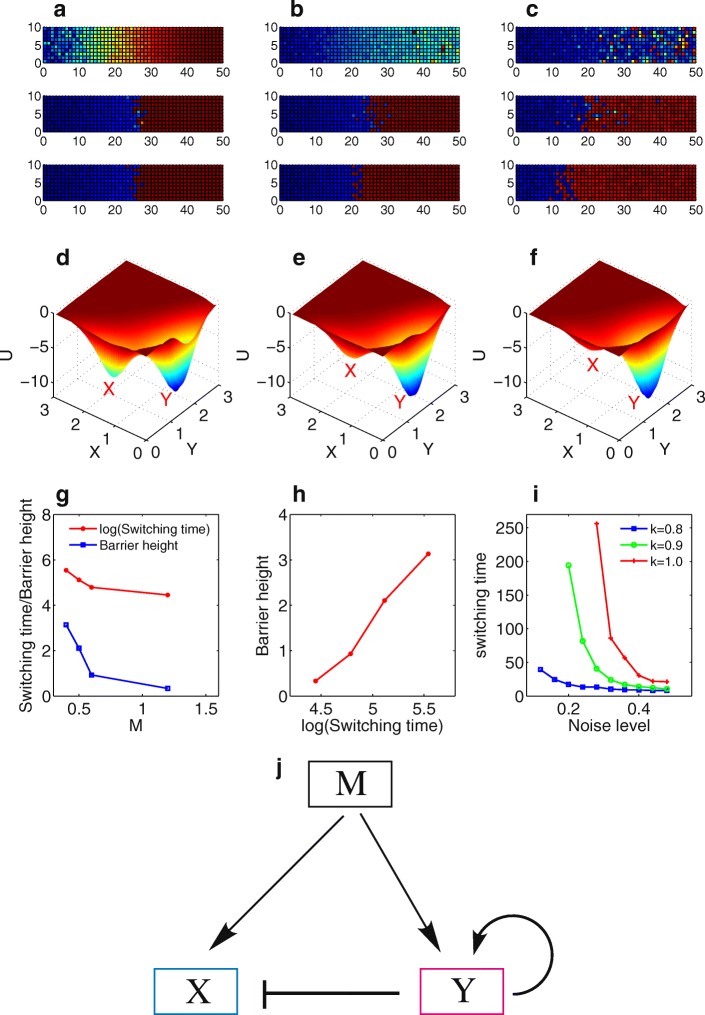
Two dimensional simulations for the boundary sharpening effects over time at different gene expression noise level *d* for the self-activation model. Blue: X is expressed, red: Y is expressed. **a** for *d* = 0, **b** for *d* = 0.01, **c** for *d* = 0.02. **d**-**f** Landscape at different morphogen level (M = 0.4 for **d**, M = 0.6 for **e**, and M = 1.2 for **f**) for the self-activation model. **g** Switching time and barrier heights change with morphogen level M. **h** Barrier heights versus the switching time. **i** Average switching time versus noise level at different k. Parameter k represents the strength of self-activation. **j** Diagram for the self-activation model

**Fig. 5:**
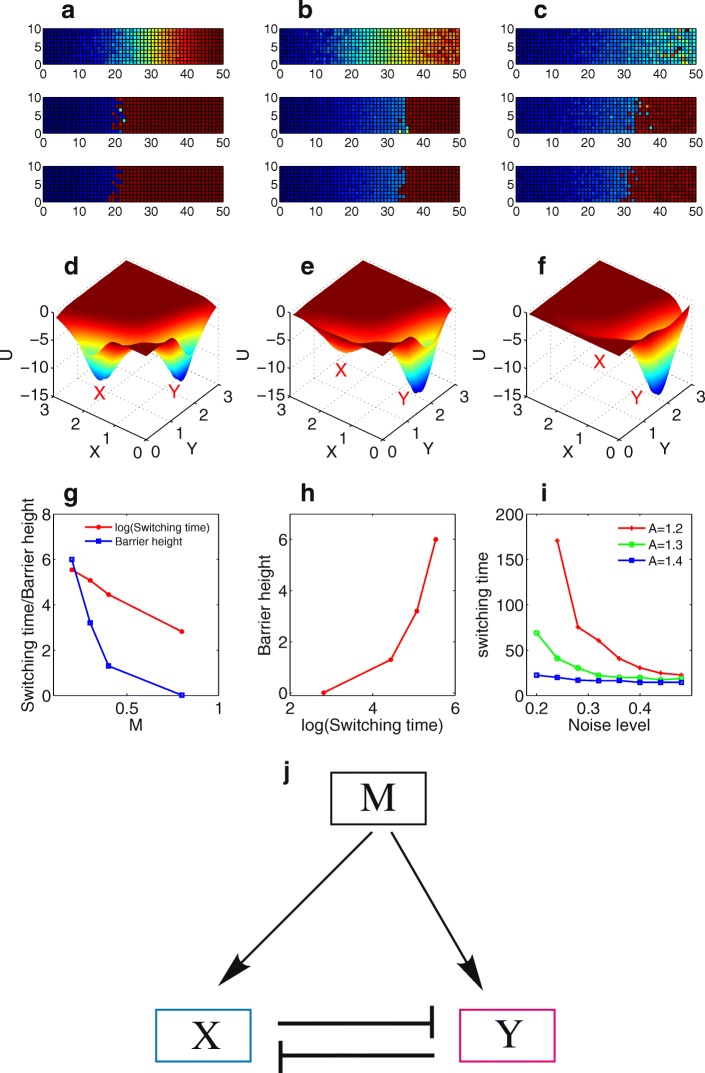
Two dimensional simulations for the boundary sharpening effects over time at different gene expression noise level *d* for the mutual repression model. Blue: X is expressed, red: Y is expressed. **a** for *d* = 0, **b** for *d* = 0.01, **c** for *d* = 0.02 **d-f** The landscape for the mutual repression (MR) model at different M level (M = 0.2 for D, M = 0.4 for E, and M = 0.8 for F). Red X and Y separately indicate the X and Y attractors. **g** Switching time and barrier heights change with morphogen level M. **h** Barrier heights versus the switching time. **i** Average switching time versus noise level for mutual repression model at different A. Parameter A represents the ratio of the synthesis rate for the two genes (gene B/gene A), which measures the degree of the asymmetry of the system. Other parameters are set as: *R* = 1, k = 1, b = 1. **j** Diagram for the mutual repression model

To explore how the boundary sharpening effects depend on the topology of the circuit, we also investigated the other two models, self-activation (SA) model (Fig. 4j) and mutual-repression (MR) model (Fig. 5j). Simulations show the boundary sharpening effects over time for the self-activation model at different noise level (Fig. 4a-c). When noise level is too low or too high, the system tends to form rough boundary (Fig. 4a and c), whereas for the medium level of noise the boundary is sharpened with time (Fig. 4b). Similar to the case for the MRSA model, the boundary sharpening effects of SA model can be explained from the probabilistic landscape perspective. With M level increased (Fig. 4d-f), the landscape changes from a relative balanced bistable state (X and Y attractor coexist) to a Y inclined bistable state, and finally to a monostable Y state. For the medium M level, the noise-induced transitions make cells switch from X state to Y state, and thus lead to the boundary sharpening. For self-activation model, a critical parameter is the self-activation strength k. We calculated the change of average switching time with noise level at different k. When the self-activation strength k is larger, the switching is faster, indicating a stronger self-activation promotes gene switching from X state to Y state, and therefore leads to a faster boundary sharpening effect.

Figure 5a-c show the boundary sharpening effects over time for the mutual-repression model (Fig. 5j) at different noise level. We also observed the boundary sharpening effects at middle level of noise from simulations (Fig. 5b). Similarly, the landscape (Fig. 5d-f) provides corresponding explanations for the boundary sharpening mechanisms.

**Fig. 6:**
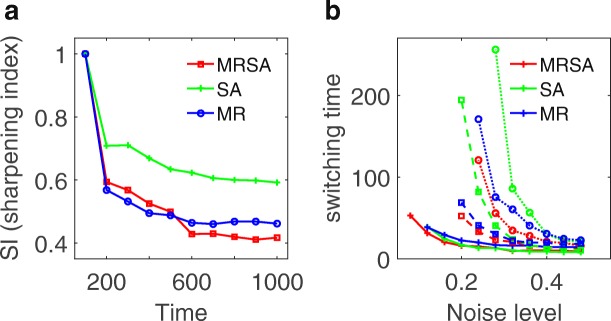
Comparisons of boundary sharpening effects and switching time for three models. **a** Comparisons of boundary sharpening effects over time quantified by sharpening index (SI), for three models. SI is the average value for multiple simulations giving parameters a fluctuation amplitude (*σ*). Here *σ* is set as 0.1. **b** The comparisons of switching times at different noise level and parameter values for the three models. Red, blue, green curves separately represent MRSA model, MR model and SA model. Three different style of curves represent three typical parameter choices. MRSA: mutual repressed self-activation model, SA: self-activation model, MR: mutual repression model

To make comparisons for three models, we define a sharpening index (SI) to quantify the boundary sharpening effects of the systems. The sharpening index is defined based on the number of the grid columns with mixed expression of X and Y around the boundary. A smaller SI means a sharper boundary. For each of the three models, we gave parameters a perturbation amplitude (σ = 0.1), and obtained an average SI from multiple simulations. Figure 6a shows the comparison results for three models. For all three models (three curves in Fig. 6a), SI decreases with time and almost reaches a steady value. This indicates all three models have the potential with the boundary sharpening ability at certain parameter regions. However, we found that the SI for the MRSA model reaches a lower value than the other two models. To validate this result, we also calculated the switching time from the landscape at different noise level and different parameter regions, and made comparisons for three models (Fig. 6b). In Fig. 6b, the red, blue, green curves represent MRSA model, MR model and SA model, separately. Three different style of curves represent three typical parameter choices for each of the three models. Although it is not easy to make comparisons directly for three models due to the limit of specific parameter choices, it appears that the MRSA model in general has the shortest switching time (the red lines are inclined to be lower than blue and green lines) among three models.

From the landscape description of the system, the boundary sharpening problem has been transformed to the understanding of state switching from X basin to Y basin, i.e. the boundary sharpening time should be roughly correlated with the state switching time between X basin and Y basin, and shorter switching time means faster boundary sharpening. Our results show that the MRSA model provides a better boundary sharpening performance than the other two models (smaller sharpening index as shown in Fig. 6a, and shorter switching time as shown in Fig. 6b). From the topological perspective, this is because MRSA motif provides a stable structure for bistability, hysteresis, and ultrasensitivity, which many biological systems employ to achieve different functions. For example, MRSA motif has been exploited in stem cell developmental system [24, 25], cancer stem cell system [23], and EMT system [26], to generate multi-stable states.

### Gene expression boundary sharpening for cross morphogen gradients

**Fig. 7:**
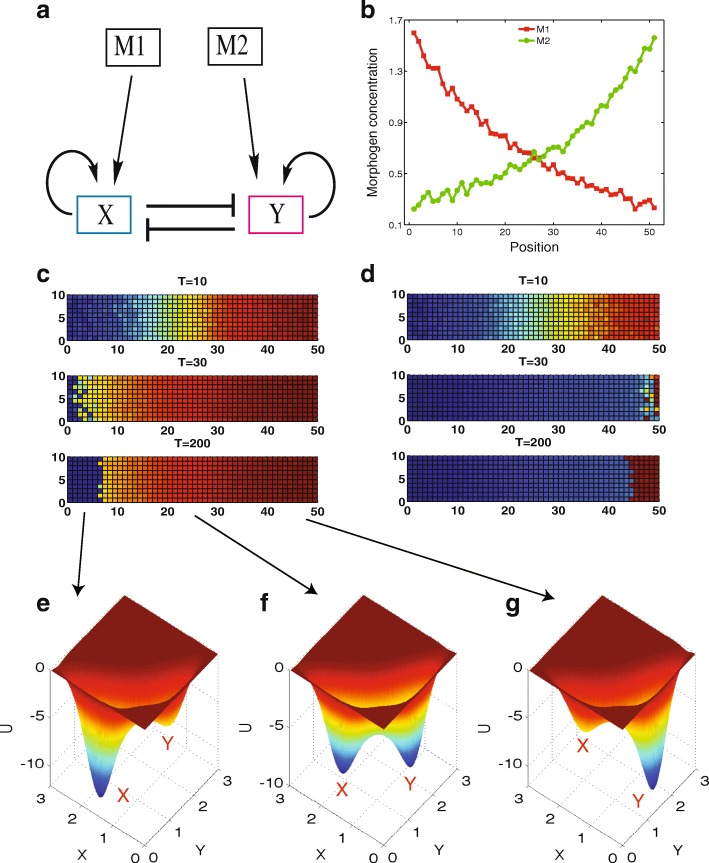
Simulations and landscapes for the MRSA model with two gradients of morphogens. **a** Diagram for the MRSA model with two gradients of morphogens. **b** Morphogen gradients for M1 and M2 with fluctuations. **c, d** Two dimensional simulations show the boundary sharpening appears in two sides of the spatial position. **e, f, g** The landscape at different position (different M1 and M2 concentration). The gradients of M1 and M2 is set as: M1 = 1, M2 = 0.38 for (**e**), M1 = 0.61, M2 = 0.61 for (**f**), and M1 = 0.38, M2 = 1 for (**g**)

It was proposed that cells can also respond to multiple morphogen signals. For example, a BMP-FGF interacting morphogen system was shown to be critical to the pattern formation in the development of forebrain [27]. Here we ask how such morphogen gradients influence the boundary sharpening effects. By adding a second gradient of morphogen to the MRSA model (Fig. 7a and b), we observed the boundary sharpening effects from simulations (Fig. 7c and d).

We found that the boundary sharpening can take place in two different situations (Fig. 7c and d). One of them is gene expression boundary is sharpened because of the switchings from state Y to state X (red to blue, Fig. 7c), and the other is gene expression boundary is sharpened because of the switchings from X state to Y state (blue to red, Fig. 7d). The difference of these two cases is that they have different initial conditions for X and Y expression. The mechanisms for this two-way boundary sharpening can be revealed from the landscape shown in Fig. 7e-g. The landscape in the central position (Fig. 7f) displays a bistable state, and two attractors have the similar stability, whereas for the left position (Fig. 7e) the landscape exhibits a bistable state biased to X attractor, and for the right position (Fig. 7g) the landscape exhibits a bistable state biased to Y attractor. So, if the initially Y is chosen to be more expressed then X (initial condition is chosen closer to Y attractor), the boundary will appear in a position between Fig. 7e and g, generating the boundary sharpening behavior as shown in Fig. 7c. On the contrary, if the initial condition choice is X is more expressed than Y (initial condition is chosen closer to X attractor), the boundary will appear in between Fig. 7f and g, generating the boundary sharpening behavior as shown in Fig. 7d.

For the case with single morphogen gradient, a requirement for the appearance of boundary sharpening effects is the appropriate choice of initial conditions (X needs to be more expressed than Y, Fig. 2) [15]. One advantage for the system with cross morphogen gradients is that it can trigger boundary sharpening effects regardless of the choice of initial conditions, i.e., the requirements for the initial conditions can be relaxed. Therefore, the cross morphogen system provides a more stable mechanism for the boundary sharpening of gene expression domain.

## Discussion

Cells employ morphogen gradients to control expression of different genes and form distinct spatial patterns. One key issue is how the boundary between gene expression domains is generated and sharpened. Studies suggest that morphogens interacting with downstream gene regulatory networks lead to ultrasensitivity and border formation [28–30]. We investigated such gene-morphogen interaction networks under fluctuations. Previous works showed that fluctuations, commonly regarded as detrimental to the robustness of regulatory networks, may play a critical role in pattern formation process, and appropriate noise level may promote the sharpening of gene expression boundaries [15]. Here, we aim to disclose the underlying mechanisms for noise to promote the boundary sharpening. By uncovering the probabilistic landscape of a morphogen-gene interaction network, we found that as the morphogen level M increases, the landscape changes from a relative balanced bistable state to a biased bistable state, and finally to a monostable state. The boundary appears in the position corresponding to the biased bistable landscape. Starting from the less stable state of the bistable states, noise will induce the state transition from the less stable state to the relative stable state. This provides a natural explanation for the noise-induced boundary sharpening mechanism.

From the analysis for the boundary sharpening ability of three circuits, we propose that to possess the boundary sharpening potential for a gene regulatory circuit with a single morphogen gradient, three conditions need to be met:

1. The system needs to form a bistable state system.
2. The bistable state needs to be asymmetric, meaning that one of the two stable states (say X state) is less stable than the other stable state (say Y state).
3. Gene X (corresponding to the less stable state) needs to be expressed initially, i.e. the initial condition should be chosen at a position closer to X attractor rather than Y attractor.

As can be seen, some basic gene regulatory motifs such as MRSA, SA, and MR, are all able to generate bistable states with appropriate parameter choices. So, the condition 1 is easy to be satisfied. However, only bistability is not sufficient to trigger boundary sharpening for the system. Only if the bistability is inclined to one of the two states (parameters need to be tuned), i.e. one of the two states is very unstable, the noise-induced switchings take place and lead to the boundary sharpening for the pattern formation system.

To explore the influence of the topology on the boundary sharpening ability of the network, we compared the boundary sharpening ability for three models: mutual repression with self-activation model, self-activation model, and mutual repression model. We define the sharpness index (SI) to quantify the boundary sharpening ability of the circuit. We found that MRSA model possesses the most stable boundary sharpening ability against parameter variations among the three models, which is supported by the results of average switching time indicating that the MRSA model has the shortest average switching time among three models. This might be because, compared to mutual repression or self-activation, MRSA model is more robust to generate bistable states or multiple states under perturbations, and thus possesses better boundary sharpening abilities. We also introduced a second morphogen gradient to explore the effects of cross morphogen gradients on the boundary sharpening of the system. We found that in the cross morphogen gradients model the boundary sharpening can appear independent on the initial concentrations of gene X and gene Y, because of a two-way switching mechanism. For single gradient model, in order to induce the boundary sharpening, gene X has to be expressed more than gene Y. Therefore, adding second gradient can make the boundary sharpening behavior more universal, providing a more stable mechanism for the sharpening of gene expression boundary.

As suggested by recent studies, the gene expression boundaries are formed and sharpened in vivo [31–33]. Our results provide possible explanations for these observations from a perspective of noise-induced cell fate switching, and insights into how noise utilizes simple gene regulatory network to perform meaningful biological functions. Our unpublished work on the formation of the cortex-dorsal midline border in the developing telencephalon suggests that the mutually inhibitory BMP and FGF signals can lead to the boundary refinement. This study provides a simple explanation on the purpose of such two-gradient system in boundary sharpening.

Our results help elucidate the critical network structures for noise attenuation and pattern formation in development. Our method is general, and can be applied to other pattern formation or spatial relevant biological systems. With more biological regulatory data available, some more realistic morphogen-gene regulatory networks can be constructed. We anticipate that, by investigating these more realistic networks, more intricate mechanisms for the boundary formation and sharpening can be discovered. This will further our understanding of boundary sharpening, pattern formation, and other spatial related issues.

## Conclusions

In this study, we discovered the probabilistic landscape for three motifs (MRSA, SA, and MR), interacting with morphogens, and investigated their boundary sharpening effects. The landscape results, along with the average switching time between attractors, provide a natural explanation for the boundary sharpening behavior depending on the noise-induced gene state switching. The MRSA model displays more robust boundary sharpening ability against parameter perturbation, compared to the MR or SA model. This is supported by the results of switching time between attractors, because the MRSA model has shortest switching time among three models. In addition, introducing cross gradients of morphogens provides a more stable mechanism for the boundary sharpening of gene expression, due to a two-way switching mechanism. Our results reveal the critical network structures for noise-induced boundary sharpening effects of gene expression, and promote the understanding for the critical network structures of spatial pattern formation in embryonic development.

## Methods

From the topology of the network for three models, we construct dynamic models to describe the temporal evolution for the expression level of different genes in the network. We first construct an ordinary differentiation equation (ODE) model based on Hill cooperativity form representing activation or repression [22]. The ODE model include three terms: basal synthesis rates, activation or repression regulations from other genes, and self-degradations. The three models (parameters can be found in Additional file 1: Tables S1-S4) are represented by the following equations:

1. The mutual repressed self-activation model (MRSA)

$$ \frac{dX}{dt}=a\left(\frac{b}{A}+\frac{X^n}{S^n+{X}^n}\right)\left(1-R+R\frac{S^n}{S^n+{Y}^n}\right)+a1\frac{M^n}{S^n+{M}^n}- kX $$

1 $$ \frac{dY}{dt}=a\left(b+\frac{Y^n}{S^n+{Y}^n}\right)\left(1-R+R\frac{S^n}{S^n+{X}^n}\right)+a1\frac{M^n}{S^n+{M}^n}- kY $$

2. The self-activation model (SA)

$$ \frac{dX}{dt}= ab1\left(1-R+R\frac{S^n}{S^n+{Y}^n}\right)+a1\frac{M^n}{S^n+{M}^n}- kX $$

2 $$ \frac{dY}{dt}=a\left(b2+\frac{Y^n}{S^n+{Y}^n}\right)+a1\frac{M^n}{S^n+{M}^n}- kY $$

3. Mutual-repression model (MR)

$$ \frac{dX}{dt}=a\frac{b}{A}\left(1-R+R\frac{S^n}{S^n+{Y}^n}\right)+a1\frac{M^n}{S^n+{M}^n}- kX $$

3 $$ \frac{dY}{dt}= ab\left(1-R+R\frac{S^n}{S^n+{X}^n}\right)+a1\frac{M^n}{S^n+{M}^n}- kY $$

4. The mutual repressed self-activation model with cross morphogen gradients

$$ \frac{dX}{dt}=a\left(\frac{b}{A}+\frac{X^n}{S^n+{X}^n}\right)\left(1-R+R\frac{S^n}{S^n+{Y}^n}\right)+a1\frac{M{1}^n}{S^n+M{1}^n}- kX $$

4 $$ \frac{dY}{dt}=a\left(b+\frac{Y^n}{S^n+{Y}^n}\right)\left(1-R+R\frac{S^n}{S^n+{X}^n}\right)+a1\frac{M{2}^n}{S^n+M{2}^n}- kY $$

Here, the ODE systems describe the temporal evolution of expression levels of X and Y genes. *S* represents the threshold of the sigmoidal function, and *n* is the Hill coefficient, which determines the steepness of the sigmoidal function [22]. The parameter *A* represents the ratio of the synthesis rate for the two genes, which measures the asymmetry level of the system. The parameter *R* represents the strength of the mutual inhibition between gene X and Y. In addition, *a* is the basal synthesis rate and *k* is the degradation rate for gene X and Y (see Additional file 1 for the descriptions of parameters, and Table S1 for the values of parameters). Take Eq. (1) as an example, the first term represents the regulation effect from gene X and Y, the second term represents the regulations from morphogen level M, and the last term represents the degradation of gene X or Y.

To generate the morphogen gradient with fluctuations, we used the model from [15], and made the spatial simulations (Eq. 5). Here [*RA*]_*out*_ and [*RA*]_*in*_ denote separately extracellular and intracellular RA concentrations. ${\left[ RA\right]}_{out}\frac{{\mathit{\partial}}^2{W}_{out}\left(t,x\right)}{\mathit{\partial t\partial x}}$ and ${\left[ RA\right]}_{in}\frac{{\mathit{\partial}}^2{W}_{out}\left(t,x\right)}{\mathit{\partial t\partial x}}$ represent standard white noise for extracellular and intracellular RA concentrations. *RA*_*in*_ is the morphogen *M* we used in the simulations, and *ϵ* quantifies the noise level in morphogen *M*. For spatial simulations, no flux boundary conditions are applied along the y axis (medial-lateral direction). Along the x axis, a no-flux boundary condition is applied at the anterior margin and a leaky boundary condition is used at the posterior margin on the assumption that extracellular RA only leaves the embryo by diffusing through cells. We have varied *ϵ* to see it’s influence on the simulation results.

$$ \frac{\mathit{\partial}{\left[ RA\right]}_{out}}{\mathit{\partial t}}={D}_{RA}\frac{{\mathit{\partial}}^2{\left[ RA\right]}_{out}}{\mathit{\partial}{x}^2}+{V}_{RA}\left(x,t\right)-\left(1+\beta \right){k}_A{\left[ RA\right]}_{out}+{k}_A{\left[ RA\right]}_{in}+{\epsilon}_{out}{\left[ RA\right]}_{out}\frac{{\mathit{\partial}}^2{W}_{out}\left(t,x\right)}{\mathit{\partial t\partial x}} $$

5 $$ \frac{\mathit{\partial}{\left[ RA\right]}_{in}}{\mathit{\partial t}}={k}_A{\left[ RA\right]}_{out}-\left({k}_A+\left[ Cyp\left({\left[ RA\right]}_{in}\right)\right]\right){\left[ RA\right]}_{in}+\epsilon {\left[ RA\right]}_{in}\frac{{\mathit{\partial}}^2{W}_{out}\left(t,x\right)}{\mathit{\partial t\partial x}} $$

For the simulations from stochastic ODEs, the initial conditions are specified as (X0, Y0) = (0.5+ sN(0,1), 0.01), as we require the system starts from a location close to X attractor, i.e. X is much larger than Y. Here N is a random variable obeying standard norm distribution, and s = 0.05 is the magnitude for the fluctuations. For each model, we ran multiple simulations (100 times) to get the average results (Figs. 1, 4, 5 and 7).

### Probabilistic landscape

In the cells, there exist intrinsic noise from statistical fluctuations of the finite number of molecules, and external noise from highly dynamical and inhomogeneous environments. Both of them can be significant to the dynamics of the system [11–13]. Therefore, one needs to study the cellular network dynamics in fluctuating conditions in order to model the cellular inner and outer environments realistically. The dynamics of a gene network in fluctuating environments can be addressed by: $\dot{x}=\mathbf{F}(x)+d\ast \mathbf{x}\ast \zeta$, where **x** = (*x*_1_(*t*), *x*_2_(*t*), …, *x*_*n*_(*t*)) represents the vector of gene expression levels. **F**(x) is the vector for the driving force of gene regulations. ζ is Gaussian noise term satisfied with: <ζ_*i*_(*x*, *t*) >  = 0 and <ζ_*i*_(*x*, *t*)ζ_*j*_(*x*, *t*^′^) >  = 2*D*_*ij*_*δ*_*ij*_*δ*(*t* − *t*^′^) (*δ*_*ij*_ = 1 for *i* = *j*, and *δ*_*ij*_ = 0 for *i* ≠ *j*), where *δ*(*t*) is Dirac delta function and *D* is diffusion coefficient matrix. Here, d is the amplitude for white noise. So, the above ODEs (Eqs. 1, 2 and 3) can be transformed to stochastic ordinary differential equations (SODE).

The probability evolution for a stochastic dynamical system can be captured by the diffusion equations [34, 35]. For a 2-dimensional system, the diffusion equation has the form:

6 $$ \frac{\mathit{\partial P}\left({x}_1,{x}_2,t\right)}{\mathit{\partial t}}=-\frac{\mathit{\partial}}{\mathit{\partial}{x}_1}\left[{F}_1\left({x}_1,{x}_2\right)P\right]-\frac{\mathit{\partial}}{\mathit{\partial}{x}_2}\left[{F}_2\left({x}_1,{x}_2\right)P\right]+D\left(\frac{{\mathit{\partial}}^2P}{\mathit{\partial}{x}_1^2}+\frac{{\mathit{\partial}}^2P}{\mathit{\partial}{x}_2^2}\right) $$

Here, *F*_1_and *F*_2_ represent the driving force for the system from above ODEs. *D* is the diffusion coefficient matrix.

By solving the diffusion equations for the long time limit, we obtained the steady state probabilistic distribution of the system. We used COMSOL Multiphysics (version 4.3) to solve the diffusion equations. In this way, we mapped out the potential landscape for the system by *U* =  − ln(*P*_*ss*_) [17, 18, 22, 23, 36]. Here, *P*_*ss*_ represents the probability distribution of the steady state, and *U* is the dimensionless potential.

We calculated the mean first passage time (MFPT) from the temporal trajectories of the variable X and Y. Starting from a random initial state at X attractor, following the temporal evolution of the system trajectory, we are able to find the time when the system first switches to the Y attractor. The time difference between initial time and the final time is defined as the first passage time (FPT) for the transition process from X attractor to Y attractor. Repeating this process we can obtain the average of the FPT, defined as the MFPT for the transition process from X attractor to Y attractor.

## Additional file

Additional file 1: **Figure S1.** Comparisons of boundary sharpening effects over time quantified by sharpening index (SI), for MRSA models at different morphogen noise level *ϵ* and gene expression noise level *d* value. **Figure S2.** Two dimensional simulations show the boundary sharpening effects over time at different vertical resolution (A for 10 grids and B for 20 grids). Blue: X is expressed, red: Y is expressed. **Table S1.** Parameters of the mutual repressed self-activation (MRSA) model. **Table S2.** Parameters of the self-activation (SA) model. **Table S3.** Parameters of the mutual repression (MR) model. **Table S4.** Parameters of the cross morphogen gradients model. (PDF 3832 kb)

## Abbreviations

FPT: First passage time
MFPT: Mean first passage time
MR: Mutual-repression
MRSA: Mutual repressed self-activation
SA: Self-activation
