# Supplementary material for: Landscape reveals critical network structures for sharpening gene expression boundaries
Source: BMC Syst Biol. 2018 Jun 13;12:67. doi: 10.1186/s12918-018-0595-5 (PMC6001026; doi:10.1186/s12918-018-0595-5)
Supplement: Supplementary file 1 — Figure S1. Comparisons of boundary sharpening effects over time quantified by sharpening index (SI), for MRSA models at different morphogen noise level ϵ and gene expression noise level d value. Figure S2. Two dimensional simulations show the boundary sharpening effects over time at different vertical resolution (A for 10 grids and B for 20 grids). Blue: X is expressed, red: Y is expressed. Table S1. Parameters of the mutual repressed self-activation (MRSA) model. Table S2. Parameters of the self-activation (SA) model. Table S3. Parameters of the mutual repression (MR) model. Table S4. Parameters of the cross morphogen gradients model. (PDF 3832 kb) [file 12918_2018_595_MOESM1_ESM.pdf]

Supporting information for: Landscape reveals critical network structures for sharpening gene expression boundaries

Chunhe Li, Lei Zhang, Qing Nie

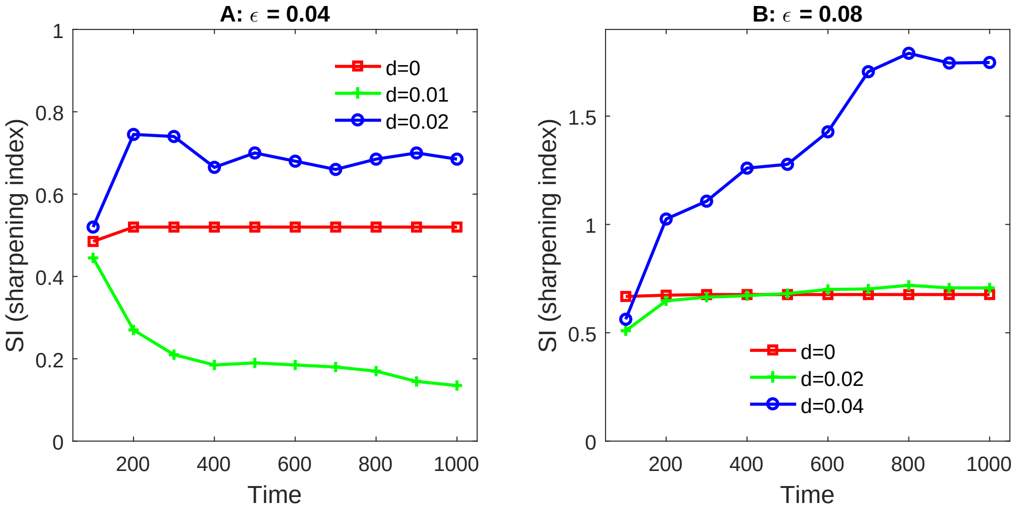

Figure S1. Comparisons of boundary sharpening effects over time quantified by sharpening index (SI), for MRSA models at different morphogen noise level ϵ and gene expression noise level d value. (A) for different d at $\epsilon = 0.04$ and (B) for different d at $\epsilon = 0.08$. For each choice of d and ϵ (each line in the figure), we run the simulations 100 times and get the average for SI. These results show that boundary sharpening effects only appear in the appropriate range for both morphogen noise level ϵ and gene expression noise level d value (green line in (A)).

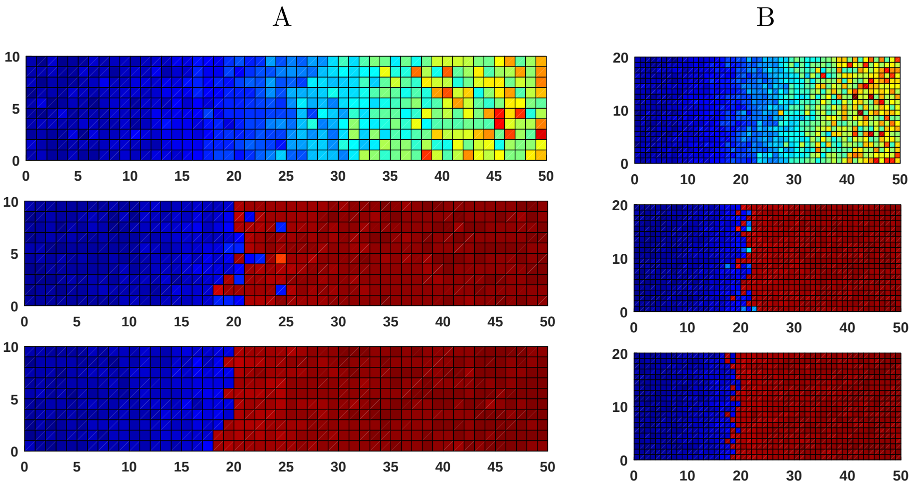

Figure S2. Two dimensional simulations show the boundary sharpening effects over time at different vertical resolution (A for 10 grids and B for 20 grids). Blue: X is expressed, red: Y is expressed.

Table S1. Parameters of the mutual repressed self-activation (MRSA) model

Symbol	Description	Value
n	Hill coefficient	4
S	threshold for Hill function	0.5
R	Repression strength	0.6
k	Degradation	1
a	Synthesis rate	0.7
b	Basal synthesis rate for X and Y	0.7
a1	Morphogen activation strength	0.1
A	ratio of the basal synthesis rate between X and Y	1.2

Table S2. Parameters of the self-activation (SA) model

Symbol	Description	Value
n	Hill coefficient	4
S	threshold for Hill function	0.5
R	Repression strength	1
k	Degradation	1
a	Synthesis rate	1
b1	Basal synthesis rate for X	1
b2	Basal synthesis rate for Y	0.1
a1	Morphogen activation strength	0.1

Table S3. Parameters of the mutual repression (MR) model

Symbol	Description	Value
n	Hill coefficient	4
S	threshold for Hill function	0.5
R	Repression strength	1
k	Degradation	1
a	Synthesis rate	1
b	Basal synthesis rate for X	1
a1	Morphogen activation strength	0.1
A	ratio of the basal synthesis rate between X and Y	1.2

Table S4. Parameters of the cross morphogen gradients model

Symbol	Description	Value
n	Hill coefficient	4
S	threshold for Hill function	0.5
R	Repression strength	0.6
k	Degradation	1
a	Synthesis rate	0.7
b	Basal synthesis rate for X	0.7
a1	Morphogen activation strength	0.1
A	ratio of the basal synthesis rate between X and Y	1
